# Extreme heat stress in older adults: A punch to the gut, kidneys or more?

**DOI:** 10.1113/EP092340

**Published:** 2024-10-27

**Authors:** Christopher L. Chapman, Zachary J. Schlader

**Affiliations:** ^1^ United States Army Research Institute of Environmental Medicine (USARIEM) Natick Massachusetts USA; ^2^ Oak Ridge Institute for Science and Education (ORISE) Oak Ridge Tennessee USA; ^3^ Department of Kinesiology Indiana University School of Public Health Bloomington Indiana USA

**Keywords:** AKI, gastrointenstinal, heat wave, kidney function

1

Older adults aged ≥65 years exposed to heat waves are particularly vulnerable to heat injury, which is characterized by hyperthermia (i.e., increased core temperature) and evidence of end‐organ damage (e.g., kidneys, gastrointestinal tract, liver) in the absence of heat stroke. While heat illness exists on a continuum, ranging from heat exhaustion to heat stroke, heat injury is a form of heat illness of moderate‐to‐high severity that is less recognized despite strong epidemiological evidence of its adverse health effects. For example, the top causes of hospitalizations in older adults during heat waves are related to the kidneys (acute kidney injury (AKI) and fluid and electrolyte disturbances) (Chapman et al., 2021), indicating that heat injury is an important public health concern.

The pathophysiology of heat injury is complex and integrated, likely stemming from hyperthermia‐induced reductions in blood flow to the splanchnic vascular beds causing ischaemia, oxidative stress and gastrointestinal hyperpermeability (Meade et al., 2020). Surprisingly, however, there are few experimental studies in humans that have directly examined how older age modifies heat injury risk, specifically with end‐organ damage outcomes, during heat stress. For example, McKenna, Atkins, Foster, et al. (2024) recently found that older adults had greater reductions in kidney function, indicative of AKI, compared to young adults (18–39 years) when exposed to a hot‐dry environment (47°C and 15% relative humidity) for 3 h. The older adults were more hyperthermic in this study. Thus, the primary role of older age for a given level of hyperthermia in end‐organ responses could not be determined.

In this issue of *Experimental Physiology*, McKenna, Atkins, Wallace, et al. (2025) addressed these limitations and provided further insight into the gastrointestinal and renal responses during heat stress when the magnitude of hyperthermia was matched between older and young adults. In nine older and nine young adults, the researchers used a water‐perfused suit model of controlled hyperthermia (circulating 50°C water) combined with cycling exercise at 20 W to mimic activities of daily living and control the magnitude of core and skin temperature increase until participants reached thermal tolerance. This methodological approach was a strength as it permitted assessments at the same level of hyperthermia in both age groups. The authors observed that small intestinal permeability increased in both groups and resulted in a mild inflammatory response. Moreover, older adults had higher gastroduodenal permeability compared to young adults. Reductions in kidney function (estimated glomerular filtration rate) did not differ between groups, while increases in pre‐injury phase urinary AKI biomarkers (insulin‐like growth factor‐binding protein‐7 (IGFBP7) × tissue inhibitor of metalloproteinases‐2 (TIMP‐2)) were not different between groups and there were no changes in injury phase urinary AKI biomarkers (neutrophil gelatinase‐associated lipocalin (NGAL) and kidney injury molecule‐1 (KIM‐1)).

These findings provide novel insights and stimulate new hypotheses regarding integrated mechanisms of pathophysiology during heat injury in older adults. It has been hypothesized that hyperthermia‐induced gastrointestinal hyperpermeability and the subsequent development of systemic inflammation is a key contributor underlying hyperthermia provoked AKI (Chapman et al., 2021). Under this hypothesis, hyperthermia causes gastrointestinal hyperpermeability that triggers an endotoxin‐mediated pro‐inflammatory state, which increases the susceptibility of the kidneys to nephrotoxic insults. Based on the findings of McKenna et al., it is likely that any differential hyperpermeability between younger and older adults is of gastroduodenal origins. Interestingly, however, the greater gastroduodenal permeability did not translate to a differentially elevated AKI risk in older adults. This observation can likely be explained by physiological and/or methodological reasons, both of which lead to important future research directions.

From a physiological perspective, the liver is the primary organ responsible for the breakdown of intestinal‐derived circulating endotoxins. When endotoxin release exceeds the hepatic capacity to neutralize these endotoxins, endotoxaemia ensues, which triggers activation of the immune system, stimulating a pro‐inflammatory state. While markers of circulating endotoxin concentrations were not measured, given that the systemic inflammatory response did not differ between older and younger adults, the findings of McKenna et al. suggest that endotoxin release did not exceed the capacity of the liver to remove endotoxins from the circulation in older adults. These findings highlight a key knowledge gap, as the interactive effects of heat stress and age on liver function are largely unexplored. Thus, future studies are required to better understand these interactions to identify strategies to prevent or treat heat injury, particularly in older adults.

An important methodological consideration is that, at baseline, older adults had reduced kidney function and elevations in some AKI risk biomarkers (KIM‐1) compared to young adults (McKenna et al., 2025). Despite these age‐related effects, the hyperthermia‐mediated changes in kidney function and AKI risk biomarkers did not differ between younger and older adults. To interpret these findings, it is important to note that the kidneys do not operate at their maximal filtration capacity under basal conditions. Renal functional reserve, defined as the capacity of the kidneys to acutely increase filtration, is decreased during hyperthermia in younger adults (Freemas et al., 2022). These findings suggest that utilization of functional reserve (via glomerular hyperfiltration and/or increased functional nephron utilization) provides a mechanism to buffer against profound reductions in kidney function when hyperthermic. Notably, renal functional reserve decreases with advancing age. As such, it can be speculated that, compared to the younger adults, the older adults utilized a greater proportion of their renal functional reserve during hyperthermia, which may explain why differential changes in kidney function between ages were not observed by McKenna et al. A logical next step would be to investigate how the compensatory strategy to utilize renal functional reserve during hyperthermia may differ with ageing. Older adults would presumably rely on increased glomerular hyperfiltration because the number of functional nephrons is reduced with ageing. This reliance on glomerular hyperfiltration could be problematic as glomerular hyperfiltration can independently lead to renal injury.

Altogether, McKenna et al.’s work highlights the complexity of the pathophysiology underlying heat injury in older adults exposed to extreme heat stress and spurs important thought processes for future experiments and hypotheses. Future work should consider the integrative nature of heat injury across multiple organ systems so that older adults can ultimately be better protected when exposed to extreme heat.

## AUTHOR CONTRIBUTIONS

All authors have read and approved the final version of this manuscript and agree to be accountable for all aspects of the work in ensuring that questions related to the accuracy or integrity of any part of the work are appropriately investigated and resolved. All persons designated as authors qualify for authorship, and all those who qualify for authorship are listed. The views presented herein are the private views of the authors and do not reflect the views of the United States Army or the Department of Defense.

## CONFLICT OF INTEREST

The authors declare no conflicts of interest.

## FUNDING INFORMATION

No funding was received for this work.

## References

[eph13691-bib-0001] Chapman, C. L. , Johnson, B. D. , Parker, M. D. , Hostler, D. , Pryor, R. R. , & Schlader, Z. J. (2021). Kidney physiology and pathophysiology during heat stress and the modification by exercise, dehydration, heat acclimation and aging. Temperature, 8(2), 108–159. 10.1080/23328940.2020.1826841 PMC8098077 33997113

[eph13691-bib-0002] Freemas, J. A. , Worley, M. L. , Gabler, M. C. , Hess, H. W. , McDeavitt, J. , Baker, T. B. , Johnson, B. D. , Chapman, C. L. , & Schlader, Z. J. (2022). Glomerular filtration rate reserve is reduced during mild passive heat stress in healthy young adults. American Journal of Physiology. Regulatory, Integrative and Comparative Physiology, 323(3), R340–r350. 35816723 10.1152/ajpregu.00090.2022 PMC9423723

[eph13691-bib-0003] McKenna, Z. J. , Atkins, W. , Wallace, T. , Jarrard, C. , Crandall, C. G. , & Foster, J. (2025). Gastrointestinal permeability and kidney injury risk during hyperthermia in young and older adults. Experimental Physiology, 110(1), 79–92. 10.1113/EP092204 PMC11689130 39417775

[eph13691-bib-0004] McKenna, Z. J. , Atkins, W. C. , Foster, J. , Belval, L. N. , Watso, J. C. , Jarrard, C. P. , & Crandall, C. G. (2024). Kidney Function Biomarkers During Extreme Heat Exposure in Young and Older Adults. Journal of the American Medical Association, 332(4), 333–335. 38913388 10.1001/jama.2024.9845 PMC11197017

[eph13691-bib-0005] Meade, R. D. , Akerman, A. P. , Notley, S. R. , McGinn, R. , Poirier, P. , Gosselin, P. , & Kenny, G. P. (2020). Physiological factors characterizing heat‐vulnerable older adults: A narrative review. Environment International, 144, 105909. 32919284 10.1016/j.envint.2020.105909

